# 2,5-Dimethyl-3-[4-(trifluoro­meth­oxy)anilino]­cyclo­hex-2-enone

**DOI:** 10.1107/S1600536811004338

**Published:** 2011-02-12

**Authors:** Henry North, Kwame Wutoh, M’egya K. Odoom, Pradeep Karla, Kenneth R. Scott, Ray J. Butcher

**Affiliations:** a Department of Pharmaceutical Sciences, Howard University, 2300 4th Street NW, Washington, DC 20059, USA; b Bowie High School, Bowie, MD 20715, USA; c Fork Union Military Academy, Fork Union, VA 23055, USA; d Department of Chemistry, Howard University, 525 College Street NW, Washington, DC 20059, USA

## Abstract

In the title compound, C_15_H_16_F_3_NO_2_, the dihedral angle between the benzene ring and the conjugated part of the cyclo­hexene ring is 60.00 (8)°. The non-conjugated part of the cyclohexene ring and the trifluoro­methyl group are both disordered over two sets of sites with occupancies of 0.835 (2) and 0.165 (2). In the crystal, mol­ecules are linked into chains along [010] by inter­molecular N—H⋯O hydrogen bonds. Weak inter­molecular C—H⋯O inter­actions also occur.

## Related literature

For the anti­convulsant properties of enamino­nes, see: Alexander *et al.* (2010 ▶, 2011 ▶); Edafiogho *et al.* (1992 ▶); Eddington *et al.* (2003 ▶); North *et al.* (2011 ▶); Scott *et al.* (1993 ▶, 1995 ▶). For related structures see: Alexander *et al.* (2010 ▶, 2011 ▶); North *et al.* (2011 ▶); Scott *et al.* (2006*a* ▶,*b* ▶).
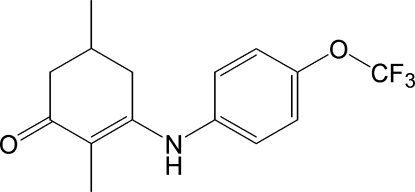


## Experimental

### 

#### Crystal data

C_15_H_16_F_3_NO_2_
*M* *_r_* = 299.29
Monoclinic, 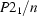

*a* = 6.10302 (11) Å
*b* = 8.39246 (16) Å
*c* = 28.2487 (5) Å
β = 93.6941 (16)°
*V* = 1443.88 (5) Å^3^
*Z* = 4
Cu *K*α radiation
μ = 1.01 mm^−1^
*T* = 123 K
0.52 × 0.36 × 0.12 mm

#### Data collection

Oxford Diffraction Xcalibur Ruby Gemini diffractometer
Absorption correction: multi-scan (*CrysAlis PRO*; Oxford Diffraction, 2009 ▶) *T*_min_ = 0.697, *T*_max_ = 1.000
5270 measured reflections
2843 independent reflections
2624 reflections with *I* > 2σ(*I*)
*R*_int_ = 0.016

#### Refinement

*R*[*F* ^2^ > 2σ(*F* ^2^)] = 0.052
*wR*(*F* ^2^) = 0.138
*S* = 1.05
2843 reflections
219 parameters
48 restraints
H-atom parameters constrained
Δρ_max_ = 0.65 e Å^−3^
Δρ_min_ = −0.50 e Å^−3^

### 

Data collection: *CrysAlis PRO* (Oxford Diffraction, 2009 ▶); cell refinement: *CrysAlis PRO*; data reduction: *CrysAlis PRO*; program(s) used to solve structure: *SHELXS97* (Sheldrick, 2008 ▶); program(s) used to refine structure: *SHELXL97* (Sheldrick, 2008 ▶); molecular graphics: *SHELXTL* (Sheldrick, 2008 ▶); software used to prepare material for publication: *SHELXTL*.

## Supplementary Material

Crystal structure: contains datablocks I, global. DOI: 10.1107/S1600536811004338/lh5206sup1.cif

Structure factors: contains datablocks I. DOI: 10.1107/S1600536811004338/lh5206Isup2.hkl

Additional supplementary materials:  crystallographic information; 3D view; checkCIF report

## Figures and Tables

**Table 1:** Hydrogen-bond geometry (Å, °)

*D*—H⋯*A*	*D*—H	H⋯*A*	*D*⋯*A*	*D*—H⋯*A*
N1—H1⋯O2^i^	0.88	2.03	2.8538 (18)	156
C9*A*—H9*AA*⋯O2^ii^	0.99	2.58	3.428 (3)	144
C10*B*—H10*B*⋯O2^ii^	1.00	2.59	3.494 (11)	150

Symmetry codes: (i) 
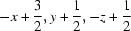
; (ii) 
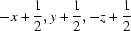
.

**Fig. 1.**
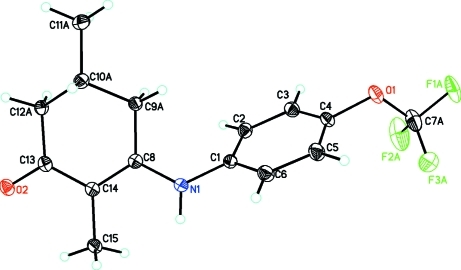
The molecular structure of the title compound. Thermal ellipsoids drawn at the 30% probability level. The disorder is not shown.

**Fig. 2.**
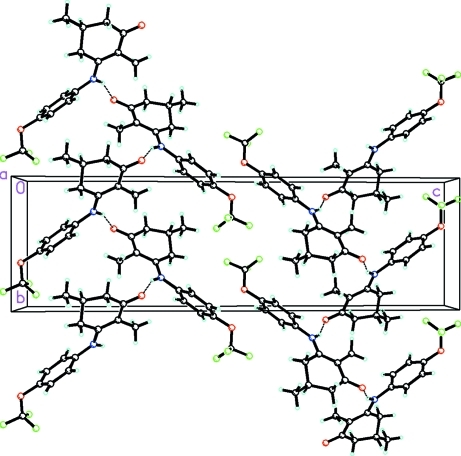
The crystal packing for 3-(4-Chlorophenylamino)-2,5-dimethylcyclohex-2-enone viewed approximately along the a axis. Hydrogen bonds are shown as dashed lines.

## supplementary crystallographic information

### Comment

The study of enaminones has led to several compounds possessing anticonvulsant properties (Edafiogho *et al.*, 1992; Eddington *et al.*, 2003; Scott *et al.*, 1993, 1995, 2006*a*,*b*; Alexander *et al.*, 2010, 2011; North *et al.*, 2011). Our group has extensively studied the effects of modification of the enaminone with substitutions at the methyl ester, ethyl ester, and without the ester group. We recently synthesized a series of methyl-substituted enaminones. The title compound, 3-(4-(trifluoromethoxy)phenylamino)-2,5-dimethylcyclohex-2-enone was exclusively active in the maximal electroshock seizure evaluation (MES) in mice, indicative of protection against tonic-clonic convulsions in humans. The MES test with mice revealed no activity at the 30 mg kg^-1^ dose, however in the 100 mg kg^-1^ dose, 1/3 of the animals were protected at 30 minutes and 3/3 of the animals were protected at 4 h. At a dose of 300 mg kg^-1^, 1/1 animals were protected at 30 min and 4 h. In the rat (po) MES study, at a dose of 30 mg kg^-1^, 2/4 of the animals were protected at 4 h with no toxicity. In the 6 Hz seizure study in mice, at a dose of 75 mg kg^-1^, 1/4 animals were protected at 30 min, 1 h, and 2 h.

Since the shape of the molecule is important in determining binding to the receptor sites it is of interest to note that the dihedral angle between the phenyl ring and the conjugated part of the cyclohexene ring is 60.00 (8)°. The backbone of the cyclohexene and the trifluoromethyl groups are disordered over two conformations with occupancies of 0.835 (2) and 0.165 (2), respectively. The geometry of the trifluoromethyl groups are idealized. The molecules are linked into chains along [010] by intermolecular N—H···O hydrogen bonds (see Fig. 2). In addition there are weak intermolecular C—H···O interactions.

### Experimental

Iodomethane (11.2 ml, 0.18 mol, 1.5 equiv) was added to a solution of 5-methyl-1,3-cyclohexanedione (15.0 g, 0.119 mol) in 4 N aqueous sodium hydroxide (30 mL, 1.0 equiv of NaOH) in a two-neck 250 ml round bottom flask fitted with a magnetic stirrer and condenser. The solution was refluxed for 20 h and cooled to room temperature, then refrigerated at 273K overnight. Vacuum filtration of the reaction mixture gave a crystalline mass dried to yield 9.24 g (54%). The crystalline mass, 2,5-dimethyl-1,3-cyclohexadione (2.10 g, 15 mmol), mp 443-445K (lit. mp 403-404.5K), 4-trifluromethoxyaniline (2.412 g, 18 mmol), and toluene (60 ml) was added to a 150 ml single neck round bottom flask containing a stir bar. The solution was refluxed and stirred for 6 h with azeotropic removal of water by Dean-Stark trap. After standing overnight, crystals appeared. Evaporation under reduced pressure yielded crystals that were recrystallized from EtOAc, 23.6% yield (mp 446-448K).

### Refinement

H atoms were placed in geometrically idealized positions and constrained to ride on their parent atoms with a C—H distance of 0.93 and 0.98 Å *U*_iso_(H) = 1.2*U*_eq_(C) and 0.96 Å for CH_3_ [*U*_iso_(H) = 1.5*U*_eq_(C)]. The H atoms attached to N were idealized with an N–H distance of 0.86 Å. The backbone of the cyclohexene and the trifluoromethyl groups were disordered over two conformations with occupancies of 0.835 (2) and 0.165 (2), respectively. The trifluoromethyl groups were idealized.

### Crystal data

**Table:**

C_15_H_16_F_3_NO_2_	*F*(000) = 624
*M**_r_* = 299.29	*D*_x_ = 1.377 Mg m^−^^3^
Monoclinic, *P*2_1_/*n*	Cu *K*α radiation, λ = 1.54184 Å
Hall symbol: -P 2yn	Cell parameters from 4019 reflections
*a* = 6.10302 (11) Å	θ = 4.7–74.0°
*b* = 8.39246 (16) Å	µ = 1.01 mm^−^^1^
*c* = 28.2487 (5) Å	*T* = 123 K
β = 93.6941 (16)°	Plate, colorless
*V* = 1443.88 (5) Å^3^	0.52 × 0.36 × 0.12 mm
*Z* = 4	

### Data collection

**Table:**

Oxford Diffraction Xcalibur Ruby Gemini diffractometer	2843 independent reflections
Radiation source: Enhance (Cu) X-ray Source	2624 reflections with *I* > 2σ(*I*)
graphite	*R*_int_ = 0.016
Detector resolution: 10.5081 pixels mm^-1^	θ_max_ = 74.2°, θ_min_ = 5.5°
ω scans	*h* = −4→7
Absorption correction: multi-scan (*CrysAlis PRO*; Oxford Diffraction, 2009)	*k* = −9→10
*T*_min_ = 0.697, *T*_max_ = 1.000	*l* = −34→34
5270 measured reflections	

### Refinement

**Table:**

Refinement on *F*^2^	Primary atom site location: structure-invariant direct methods
Least-squares matrix: full	Secondary atom site location: difference Fourier map
*R*[*F*^2^ > 2σ(*F*^2^)] = 0.052	Hydrogen site location: inferred from neighbouring sites
*wR*(*F*^2^) = 0.138	H-atom parameters constrained
*S* = 1.05	*w* = 1/[σ^2^(*F*_o_^2^) + (0.0709*P*)^2^ + 0.9652*P*] where *P* = (*F*_o_^2^ + 2*F*_c_^2^)/3
2843 reflections	(Δ/σ)_max_ < 0.001
219 parameters	Δρ_max_ = 0.65 e Å^−^^3^
48 restraints	Δρ_min_ = −0.50 e Å^−^^3^

### Special details

**Table:**

Geometry. All e.s.d.'s (except the e.s.d. in the dihedral angle between two l.s. planes) are estimated using the full covariance matrix. The cell e.s.d.'s are taken into account individually in the estimation of e.s.d.'s in distances, angles and torsion angles; correlations between e.s.d.'s in cell parameters are only used when they are defined by crystal symmetry. An approximate (isotropic) treatment of cell e.s.d.'s is used for estimating e.s.d.'s involving l.s. planes.
Refinement. Refinement of *F*^2^ against ALL reflections. The weighted *R*-factor *wR* and goodness of fit *S* are based on *F*^2^, conventional *R*-factors *R* are based on *F*, with *F* set to zero for negative *F*^2^. The threshold expression of *F*^2^ > σ(*F*^2^) is used only for calculating *R*-factors(gt) *etc*. and is not relevant to the choice of reflections for refinement. *R*-factors based on *F*^2^ are statistically about twice as large as those based on *F*, and *R*- factors based on ALL data will be even larger.

### Fractional atomic coordinates and isotropic or equivalent isotropic displacement parameters (Å^2^)

**Table:**

	*x*	*y*	*z*	*U*_iso_*/*U*_eq_	Occ. (<1)
C7A	0.7405 (3)	1.2972 (3)	0.48394 (6)	0.0462 (7)	0.835 (2)
F1A	0.7047 (3)	1.3585 (5)	0.52573 (7)	0.0656 (5)	0.835 (2)
F2A	0.6311 (4)	1.3805 (3)	0.45056 (7)	0.0799 (8)	0.835 (2)
F3A	0.9521 (3)	1.3054 (2)	0.47731 (6)	0.0656 (6)	0.835 (2)
C7B	0.6407 (12)	1.2885 (12)	0.4867 (3)	0.0462 (7)	0.165 (2)
F1B	0.4247 (12)	1.2958 (11)	0.4830 (3)	0.0656 (5)	0.165 (2)
F2B	0.7200 (17)	1.3625 (17)	0.4504 (4)	0.0799 (8)	0.165 (2)
F3B	0.7146 (16)	1.358 (3)	0.5264 (3)	0.0656 (6)	0.165 (2)
O1	0.6742 (2)	1.14194 (17)	0.48572 (4)	0.0373 (3)	
O2	0.41421 (19)	0.38222 (15)	0.21250 (4)	0.0293 (3)	
N1	0.6984 (2)	0.75651 (17)	0.32496 (5)	0.0257 (3)	
H1	0.8076	0.7746	0.3068	0.031*	
C1	0.6876 (3)	0.85474 (19)	0.36566 (6)	0.0240 (4)	
C2	0.4982 (3)	0.9395 (2)	0.37426 (6)	0.0266 (4)	
H2A	0.3718	0.9309	0.3530	0.032*	
C3	0.4944 (3)	1.0364 (2)	0.41394 (6)	0.0284 (4)	
H3A	0.3641	1.0916	0.4207	0.034*	
C4	0.6822 (3)	1.0518 (2)	0.44349 (6)	0.0275 (4)	
C5	0.8737 (3)	0.9724 (2)	0.43486 (6)	0.0319 (4)	
H5A	1.0020	0.9859	0.4553	0.038*	
C6	0.8753 (3)	0.8729 (2)	0.39589 (6)	0.0300 (4)	
H6A	1.0054	0.8164	0.3897	0.036*	
C8	0.5578 (3)	0.63770 (19)	0.31118 (6)	0.0223 (3)	
C9A	0.3906 (7)	0.5897 (3)	0.34518 (15)	0.0240 (7)	0.835 (2)
H9AA	0.2592	0.6584	0.3402	0.029*	0.835 (2)
H9AB	0.4523	0.6060	0.3781	0.029*	0.835 (2)
C10A	0.3226 (3)	0.4141 (2)	0.33854 (7)	0.0262 (4)	0.835 (2)
H10A	0.4519	0.3455	0.3481	0.031*	0.835 (2)
C11A	0.1365 (5)	0.3730 (3)	0.37008 (10)	0.0351 (6)	0.835 (2)
H11A	0.0979	0.2603	0.3661	0.053*	0.835 (2)
H11B	0.0081	0.4390	0.3611	0.053*	0.835 (2)
H11C	0.1842	0.3934	0.4033	0.053*	0.835 (2)
C12A	0.2560 (7)	0.3828 (6)	0.28575 (9)	0.0241 (7)	0.835 (2)
H12A	0.2367	0.2667	0.2809	0.029*	0.835 (2)
H12B	0.1126	0.4345	0.2776	0.029*	0.835 (2)
C9B	0.412 (5)	0.555 (3)	0.3486 (10)	0.0240 (7)	0.165 (2)
H9BA	0.4955	0.4648	0.3638	0.029*	0.165 (2)
H9BB	0.3813	0.6322	0.3737	0.029*	0.165 (2)
C10B	0.2094 (17)	0.4962 (13)	0.3272 (4)	0.0262 (4)	0.165 (2)
H10B	0.1285	0.5830	0.3091	0.031*	0.165 (2)
C11B	0.065 (3)	0.4229 (19)	0.3647 (6)	0.0351 (6)	0.165 (2)
H11D	−0.0731	0.3842	0.3490	0.053*	0.165 (2)
H11E	0.0327	0.5043	0.3881	0.053*	0.165 (2)
H11F	0.1430	0.3340	0.3806	0.053*	0.165 (2)
C12B	0.275 (5)	0.380 (3)	0.2968 (7)	0.0241 (7)	0.165 (2)
H12C	0.1424	0.3247	0.2831	0.029*	0.165 (2)
H12D	0.3638	0.3009	0.3156	0.029*	0.165 (2)
C13	0.4186 (3)	0.4425 (2)	0.25288 (6)	0.0234 (3)	
C14	0.5677 (3)	0.56830 (19)	0.26727 (5)	0.0221 (3)	
C15	0.7221 (3)	0.6296 (2)	0.23177 (6)	0.0266 (4)	
H15A	0.7160	0.7463	0.2309	0.040*	
H15B	0.6782	0.5871	0.2003	0.040*	
H15C	0.8722	0.5953	0.2411	0.040*	

### Atomic displacement parameters (Å^2^)

**Table:**

	*U*^11^	*U*^22^	*U*^33^	*U*^12^	*U*^13^	*U*^23^
C7A	0.067 (2)	0.0396 (13)	0.0315 (11)	0.0034 (14)	−0.0007 (13)	−0.0097 (10)
F1A	0.0878 (13)	0.0630 (11)	0.0457 (9)	0.0048 (10)	0.0030 (9)	−0.0356 (8)
F2A	0.133 (2)	0.0381 (10)	0.0634 (10)	0.0262 (13)	−0.0302 (13)	−0.0070 (8)
F3A	0.0776 (12)	0.0588 (11)	0.0630 (11)	−0.0308 (9)	0.0239 (9)	−0.0170 (9)
C7B	0.067 (2)	0.0396 (13)	0.0315 (11)	0.0034 (14)	−0.0007 (13)	−0.0097 (10)
F1B	0.0878 (13)	0.0630 (11)	0.0457 (9)	0.0048 (10)	0.0030 (9)	−0.0356 (8)
F2B	0.133 (2)	0.0381 (10)	0.0634 (10)	0.0262 (13)	−0.0302 (13)	−0.0070 (8)
F3B	0.0776 (12)	0.0588 (11)	0.0630 (11)	−0.0308 (9)	0.0239 (9)	−0.0170 (9)
O1	0.0491 (8)	0.0401 (8)	0.0232 (6)	−0.0027 (6)	0.0052 (5)	−0.0094 (5)
O2	0.0263 (6)	0.0351 (7)	0.0261 (6)	0.0009 (5)	−0.0013 (5)	−0.0081 (5)
N1	0.0273 (7)	0.0290 (7)	0.0215 (7)	−0.0044 (6)	0.0060 (5)	−0.0033 (6)
C1	0.0286 (8)	0.0242 (8)	0.0195 (7)	−0.0043 (6)	0.0033 (6)	0.0003 (6)
C2	0.0276 (8)	0.0274 (8)	0.0243 (8)	−0.0016 (7)	−0.0018 (6)	0.0003 (6)
C3	0.0291 (8)	0.0280 (8)	0.0286 (8)	0.0027 (7)	0.0039 (7)	−0.0010 (7)
C4	0.0350 (9)	0.0301 (9)	0.0177 (7)	−0.0026 (7)	0.0047 (6)	−0.0028 (6)
C5	0.0295 (9)	0.0416 (10)	0.0239 (8)	−0.0011 (8)	−0.0025 (6)	−0.0037 (7)
C6	0.0263 (8)	0.0366 (10)	0.0272 (9)	0.0019 (7)	0.0026 (6)	−0.0034 (7)
C8	0.0229 (7)	0.0214 (8)	0.0222 (8)	0.0019 (6)	−0.0002 (6)	0.0024 (6)
C9A	0.0312 (14)	0.0200 (19)	0.0214 (11)	−0.0004 (13)	0.0053 (10)	0.0006 (14)
C10A	0.0271 (10)	0.0247 (10)	0.0267 (10)	−0.0009 (7)	0.0015 (8)	0.0021 (8)
C11A	0.0386 (15)	0.0365 (15)	0.0309 (11)	−0.0095 (10)	0.0069 (11)	0.0020 (10)
C12A	0.0236 (12)	0.0291 (9)	0.0190 (18)	−0.0039 (8)	−0.0029 (13)	−0.0022 (15)
C9B	0.0312 (14)	0.0200 (19)	0.0214 (11)	−0.0004 (13)	0.0053 (10)	0.0006 (14)
C10B	0.0271 (10)	0.0247 (10)	0.0267 (10)	−0.0009 (7)	0.0015 (8)	0.0021 (8)
C11B	0.0386 (15)	0.0365 (15)	0.0309 (11)	−0.0095 (10)	0.0069 (11)	0.0020 (10)
C12B	0.0236 (12)	0.0291 (9)	0.0190 (18)	−0.0039 (8)	−0.0029 (13)	−0.0022 (15)
C13	0.0211 (7)	0.0247 (8)	0.0239 (8)	0.0058 (6)	−0.0020 (6)	−0.0013 (6)
C14	0.0222 (7)	0.0229 (8)	0.0210 (7)	0.0032 (6)	0.0003 (6)	0.0024 (6)
C15	0.0297 (8)	0.0272 (8)	0.0231 (8)	0.0004 (7)	0.0050 (6)	−0.0016 (6)

### Geometric parameters (Å, °)

**Table:**

C7A—F3A	1.3189 (15)	C9A—H9AB	0.9900
C7A—F1A	1.3189 (14)	C10A—C11A	1.527 (3)
C7A—F2A	1.3195 (15)	C10A—C12A	1.543 (3)
C7A—O1	1.366 (3)	C10A—H10A	1.0000
C7B—O1	1.248 (10)	C11A—H11A	0.9800
C7B—F2B	1.3169 (16)	C11A—H11B	0.9800
C7B—F1B	1.3170 (16)	C11A—H11C	0.9800
C7B—F3B	1.3171 (16)	C12A—C13	1.489 (4)
O1—C4	1.416 (2)	C12A—H12A	0.9900
O2—C13	1.246 (2)	C12A—H12B	0.9900
N1—C8	1.356 (2)	C9B—C10B	1.43 (3)
N1—C1	1.420 (2)	C9B—H9BA	0.9900
N1—H1	0.8800	C9B—H9BB	0.9900
C1—C2	1.392 (2)	C10B—C12B	1.37 (3)
C1—C6	1.392 (2)	C10B—C11B	1.550 (18)
C2—C3	1.386 (2)	C10B—H10B	1.0000
C2—H2A	0.9500	C11B—H11D	0.9800
C3—C4	1.380 (2)	C11B—H11E	0.9800
C3—H3A	0.9500	C11B—H11F	0.9800
C4—C5	1.381 (3)	C12B—C13	1.65 (3)
C5—C6	1.382 (2)	C12B—H12C	0.9900
C5—H5A	0.9500	C12B—H12D	0.9900
C6—H6A	0.9500	C13—C14	1.436 (2)
C8—C14	1.375 (2)	C14—C15	1.510 (2)
C8—C9A	1.501 (5)	C15—H15A	0.9800
C8—C9B	1.59 (3)	C15—H15B	0.9800
C9A—C10A	1.539 (4)	C15—H15C	0.9800
C9A—H9AA	0.9900		
			
F3A—C7A—F1A	109.09 (11)	C9A—C10A—C12A	109.5 (3)
F3A—C7A—F2A	109.02 (11)	C11A—C10A—H10A	108.4
F1A—C7A—F2A	109.13 (11)	C9A—C10A—H10A	108.4
F3A—C7A—O1	110.47 (16)	C12A—C10A—H10A	108.4
F1A—C7A—O1	105.8 (2)	C13—C12A—C10A	113.6 (3)
F2A—C7A—O1	113.21 (16)	C13—C12A—H12A	108.8
O1—C7B—F2B	112.3 (8)	C10A—C12A—H12A	108.8
O1—C7B—F1B	102.0 (7)	C13—C12A—H12B	108.8
F2B—C7B—F1B	109.45 (12)	C10A—C12A—H12B	108.8
O1—C7B—F3B	113.9 (10)	H12A—C12A—H12B	107.7
F2B—C7B—F3B	109.41 (13)	C10B—C9B—C8	112.0 (18)
F1B—C7B—F3B	109.45 (12)	C10B—C9B—H9BA	109.2
C7B—O1—C4	124.0 (4)	C8—C9B—H9BA	109.2
C7A—O1—C4	116.88 (13)	C10B—C9B—H9BB	109.2
C8—N1—C1	126.71 (14)	C8—C9B—H9BB	109.2
C8—N1—H1	116.6	H9BA—C9B—H9BB	107.9
C1—N1—H1	116.6	C12B—C10B—C9B	103.2 (17)
C2—C1—C6	119.79 (15)	C12B—C10B—C11B	110.3 (15)
C2—C1—N1	121.37 (15)	C9B—C10B—C11B	111.2 (14)
C6—C1—N1	118.73 (15)	C12B—C10B—H10B	110.6
C3—C2—C1	119.91 (15)	C9B—C10B—H10B	110.6
C3—C2—H2A	120.0	C11B—C10B—H10B	110.6
C1—C2—H2A	120.0	C10B—C11B—H11D	109.5
C4—C3—C2	119.17 (16)	C10B—C11B—H11E	109.5
C4—C3—H3A	120.4	H11D—C11B—H11E	109.5
C2—C3—H3A	120.4	C10B—C11B—H11F	109.5
C3—C4—C5	121.83 (16)	H11D—C11B—H11F	109.5
C3—C4—O1	119.18 (15)	H11E—C11B—H11F	109.5
C5—C4—O1	118.82 (16)	C10B—C12B—C13	116 (2)
C4—C5—C6	118.81 (16)	C10B—C12B—H12C	108.3
C4—C5—H5A	120.6	C13—C12B—H12C	108.3
C6—C5—H5A	120.6	C10B—C12B—H12D	108.3
C5—C6—C1	120.43 (16)	C13—C12B—H12D	108.3
C5—C6—H6A	119.8	H12C—C12B—H12D	107.4
C1—C6—H6A	119.8	O2—C13—C14	122.25 (15)
N1—C8—C14	120.43 (15)	O2—C13—C12A	117.27 (19)
N1—C8—C9A	117.2 (2)	C14—C13—C12A	120.44 (18)
C14—C8—C9A	122.3 (2)	O2—C13—C12B	125.3 (9)
N1—C8—C9B	120.2 (10)	C14—C13—C12B	112.2 (9)
C14—C8—C9B	118.3 (10)	C8—C14—C13	120.20 (15)
C8—C9A—C10A	111.6 (3)	C8—C14—C15	121.35 (15)
C8—C9A—H9AA	109.3	C13—C14—C15	118.29 (14)
C10A—C9A—H9AA	109.3	C14—C15—H15A	109.5
C8—C9A—H9AB	109.3	C14—C15—H15B	109.5
C10A—C9A—H9AB	109.3	H15A—C15—H15B	109.5
H9AA—C9A—H9AB	108.0	C14—C15—H15C	109.5
C11A—C10A—C9A	110.5 (2)	H15A—C15—H15C	109.5
C11A—C10A—C12A	111.5 (2)	H15B—C15—H15C	109.5
			
F2B—C7B—O1—C7A	51.0 (9)	C14—C8—C9A—C10A	29.3 (3)
F1B—C7B—O1—C7A	168.1 (11)	C9B—C8—C9A—C10A	−45 (6)
F3B—C7B—O1—C7A	−74.1 (10)	C8—C9A—C10A—C11A	−174.4 (2)
F2B—C7B—O1—C4	−32.0 (7)	C8—C9A—C10A—C12A	−51.3 (3)
F1B—C7B—O1—C4	85.1 (5)	C11A—C10A—C12A—C13	171.7 (3)
F3B—C7B—O1—C4	−157.1 (4)	C9A—C10A—C12A—C13	49.2 (4)
F3A—C7A—O1—C7B	−178.9 (9)	N1—C8—C9B—C10B	150.8 (11)
F1A—C7A—O1—C7B	63.1 (9)	C14—C8—C9B—C10B	−40.8 (17)
F2A—C7A—O1—C7B	−56.3 (9)	C9A—C8—C9B—C10B	72 (5)
F3A—C7A—O1—C4	−66.21 (18)	C8—C9B—C10B—C12B	64.6 (18)
F1A—C7A—O1—C4	175.85 (15)	C8—C9B—C10B—C11B	−177.2 (12)
F2A—C7A—O1—C4	56.4 (2)	C9B—C10B—C12B—C13	−64.6 (19)
C8—N1—C1—C2	−54.0 (2)	C11B—C10B—C12B—C13	176.6 (13)
C8—N1—C1—C6	129.73 (18)	C10A—C12A—C13—O2	158.8 (2)
C6—C1—C2—C3	−2.7 (3)	C10A—C12A—C13—C14	−23.4 (4)
N1—C1—C2—C3	−178.93 (15)	C10A—C12A—C13—C12B	16 (6)
C1—C2—C3—C4	2.4 (3)	C10B—C12B—C13—O2	−147.1 (11)
C2—C3—C4—C5	−0.5 (3)	C10B—C12B—C13—C14	37.9 (19)
C2—C3—C4—O1	−175.70 (15)	C10B—C12B—C13—C12A	−106 (7)
C7B—O1—C4—C3	−65.7 (5)	N1—C8—C14—C13	179.94 (14)
C7A—O1—C4—C3	−96.11 (19)	C9A—C8—C14—C13	−1.6 (3)
C7B—O1—C4—C5	119.0 (5)	C9B—C8—C14—C13	11.6 (10)
C7A—O1—C4—C5	88.5 (2)	N1—C8—C14—C15	−4.7 (2)
C3—C4—C5—C6	−1.1 (3)	C9A—C8—C14—C15	173.77 (17)
O1—C4—C5—C6	174.17 (16)	C9B—C8—C14—C15	−173.0 (10)
C4—C5—C6—C1	0.7 (3)	O2—C13—C14—C8	176.07 (15)
C2—C1—C6—C5	1.1 (3)	C12A—C13—C14—C8	−1.7 (3)
N1—C1—C6—C5	177.47 (16)	C12B—C13—C14—C8	−8.8 (10)
C1—N1—C8—C14	169.97 (15)	O2—C13—C14—C15	0.6 (2)
C1—N1—C8—C9A	−8.6 (3)	C12A—C13—C14—C15	−177.2 (2)
C1—N1—C8—C9B	−21.9 (10)	C12B—C13—C14—C15	175.7 (10)
N1—C8—C9A—C10A	−152.2 (2)		

### Hydrogen-bond geometry (Å, °)

**Table:**

*D*—H···*A*	*D*—H	H···*A*	*D*···*A*	*D*—H···*A*
N1—H1···O2^i^	0.88	2.03	2.8538 (18)	156
C9A—H9AA···O2^ii^	0.99	2.58	3.428 (3)	144
C10B—H10B···O2^ii^	1.00	2.59	3.494 (11)	150

Symmetry codes: (i) −*x*+3/2, *y*+1/2, −*z*+1/2; (ii) −*x*+1/2, *y*+1/2, −*z*+1/2.
